# Women Physicians in Transition Learning to Navigate the Pipeline from Early to Mid-Career: Protocol for a Qualitative Study

**DOI:** 10.2196/38126

**Published:** 2022-06-02

**Authors:** Tiffany I Leung, Karen H Wang, Tammy L Lin, Geneen T Gin, Sima S Pendharkar, Chwen-Yuen Angie Chen

**Affiliations:** 1 Care and Public Health Research Institute Maastricht University Maastricht Netherlands; 2 Department of Internal Medicine (adjunct) Southern Illinois University School of Medicine Springfield, IL United States; 3 Department of Internal Medicine Yale School of Medicine New Haven, CT United States; 4 Center for Medical Informatics Yale School of Medicine New Haven, CT United States; 5 Department of Medicine (voluntary) University of California San Diego Health Sciences San Diego, CA United States; 6 Department of Family Medicine and Public Health University of California San Diego School of Medicine La Jolla, CA United States; 7 Division of Hospital Medicine Jersey City Medical Center Jersey City, NJ United States; 8 Department of Primary Care and Population Health Stanford University Palo Alto, CA United States

**Keywords:** gender equity, women physician, female physicians, career development, professional development, career pipeline, leaky pipeline, mid-career physicians, early-career physicians, physician, healthcare profession, peer support, physician perspective, physician experience, professional learning, healthcare, health care, healthcare education, career support, gender equality, gender bias, healthcare learning

## Abstract

**Background:**

Women physicians face unique obstacles while progressing through their careers, navigating career advancement and seeking balance between professional and personal responsibilities. Systemic changes, along with individual and institutional changes, are needed to overcome obstacles perpetuating physician gender inequities. Developing a deeper understanding of women physicians’ experiences during important transition points could reveal both barriers and opportunities for recruitment, retention, and promotion, and inform best practices developed based on these experiences.

**Objective:**

The aim is to learn from the experiences and perspectives of women physicians as they transition from early to mid-career, then develop best practices that can serve to support women physicians as they advance through their careers.

**Methods:**

Semistructured interviews were conducted with women physicians in the United States in 2020 and 2021. Eligibility criteria included self-identification as a woman who is in the process of transitioning or who recently transitioned from early to mid-career stage. Purposeful sampling facilitated identification of participants who represented diversity in career pathway, practice setting, specialty, and race/ethnicity. Each participant was offered compensation for their participation. Interviews were audio-recorded and professionally transcribed. Interview questions were open-ended, exploring participants' perceptions of this transition. Qualitative thematic analysis will be performed. We will use an open coding and grounded theory approach on interview transcripts.

**Results:**

The Ethics Review Committee of the Faculty of Health, Medicine, and Life Sciences at Maastricht University approved the study; Stanford University expedited review approved the study; and the University of California, San Diego certified the study as exempt from review. Twelve in-depth interviews of 50-100 minutes in duration were completed. Preliminary analyses indicate one key theme is a tension resulting from finite time divided between demands from a physician career and demands from family needs. In turn, this results in constant boundary control between these life domains that are inextricable and seemingly competing against each other within a finite space; family needs impinge on planned career goals, if the boundary between them is not carefully managed. To remedy this, women sought resources to help them redistribute home responsibilities, freeing themselves to have more time, especially for children. Women similarly sought resources to help with career advancement, although not with regard to time directly, but to first address foundational knowledge gaps about career milestones and how to achieve them.

**Conclusions:**

Preliminary results provide initial insights about how women identify or activate a career shift and how they marshaled resources and support to navigate barriers they faced. Further analyses are continuing as of March 2022 and are expected to be completed by June 2022. The dissemination plan includes peer-reviewed open-access journal publication of the results and presentation at the annual meeting of the American Medical Association’s Women Physicians Section.

## Introduction

### Career Advancement Disparities

As women physicians transition through different stages of their careers, their professional skills and personal or family obligations may be evolving in parallel. For example, physicians can transition from early to mid-career as a leader, executive, educator, researcher, advocate, entrepreneur, or a combination thereof. These women, often in their 30s and 40s, are therefore experiencing peak potential for the need to make choices between their professional and personal worlds. The leaky pipeline of women physicians advancing through their careers remains problematic, and focused investigation of important inflection points, such as the transition from early to mid-career, could contribute important insights to our understanding of how to achieve gender equity in medical careers.

The medical profession has achieved significant progress in understanding gender inequity and its consequences [1,2], yet actualized gender equity has not yet been reached in any career pathway for women physicians. Women make up nearly half of US medical school applicants [3,4], and more than half of the physician workforce in certain specialties. In US academia, women make up only 38% of faculty, 21% of full professors, and 16% of deans [3]. Lack of gender equity is also a concern in international academic medical settings, where women clinician educators are less likely to hold academic appointments, and are more likely to be younger, single, and childless [5,6]. Although the number of women at all levels of academic medicine has increased [7], balancing an academic career with raising a family is hard work for women physicians [8]. Women faculty tend to leave an institution because of a lack of professional advancement, low salary, or departmental leadership issues [9].

Women are affected in all other career pathways, in addition to the academic pipeline. Far fewer women in general, including women physicians, hold chief executive positions compared to male physicians and men in general [5,10], and women executives of hospitals are often paid less [11,12]. This may, in part, be related to the higher likelihood for women physicians to experience nonlinear career paths and they often have less access to mentors, sponsors, and leadership positions in general [13,14]. Career satisfaction for women physicians is also more likely to be influenced by work-life balance, mentorship opportunities, supportive leadership, conductive culture, and equal access [5,6,15]. Women and women physicians are also significantly underrepresented among health care startups in the male-dominated industry of health care technology [10].

### Compensation Disparities

Other barriers that women physicians face relate to gender equity in compensation [1]; advancing toward leadership in academic medicine [3,4,16], including executive positions [17]; receiving acknowledgement via medical society recognition and awards [18], and maintaining a sustainable level of engagement and fulfillment between professional life and motherhood [8] or even singlehood. Financial implications are significant for women physicians through the course of their careers [19]. Women physicians may negotiate less aggressively and with less confidence than men, or not negotiate at all, and appear to be more likely than men to take on both internal and external service [20] or volunteer for “invisible work” that is less likely to lead to promotions [21,22]. This can have financial and other significant consequences for the woman and her dependents. Among US physicians, men are paid more than women in all specialties at every academic rank; in general, this trend persists even after accounting for specialty, geography, time in practice, and average hours worked per week [23].

Some studies showed marked differences in the compensation of female physicians, which may impact their ability to support a family while pursuing a career [1]. In other cases, some female physicians are the primary breadwinners of their families and may face additional pressures or stressors associated with such a role, especially when also carrying a large share of child and family care responsibilities. Despite evidence of delivering more preventive care and better outcomes [24,25], spending more time with patients and on electronic health record tasks, and generating equal revenue after adjustments for physician, patient, and visit characteristics, women physicians are still at high risk of being undervalued in the current payment system [26].

### Work-Life Integration Disparities

Women physicians may experience an increasing need for further developing personal skills in integrating professional and family obligations (including care of young children or aging parents) [17,27-30]; skills as a mentor and sponsor of younger physicians and physician peers, or finding mentors, sponsors, and coaches to help their career advancement [13,14]; skills in negotiation and advocacy as a leader locally or on a broader scale [30]; and/or learning to better recognize their own self-care needs when facing burnout or other mental or physical health issues [31].

One study sampled male and female residents and faculty to describe physicians’ experience of work, work-family factors, and other perspectives that may influence career decision-making; emerging themes identified family obligations as being in balance or not, with motivating factors for being a physician, across a fulcrum of pursuing a career in academic internal medicine [32]. Women faculty physicians were more likely to express experiences of imbalance between their role as a practitioner and nonwork roles, compared to their counterparts who were men. Women physician practitioners also had the fewest strategies for multiple role planning compared to women researchers and educators [32]. Women physicians in transition may also face family planning challenges and infertility, in addition to these other factors [33].

Attrition during residency, for example in general surgery, has been shown to be variably influenced by female gender/sex [34-36]. In a qualitative study, female and male residents who left their general surgery training expressed an absence of a safe space for sharing program-related and personal concerns, as well as a scarcity of role models who worked in surgical careers with work-life balance that was better than the work hours of residency [34]. In addition, women resident physicians commonly strategized their second shift roles (specifically, their role as a parent), whereas men resident physicians strategized protection of personal time [32].

In addition, well-being for women physicians may have unique contributing factors [2,31], for example, women physicians experience high rates of sexism and sexual harassment [37-40], but they also face less explicit biases, such as gendered expectations of how they provided patient care [41] and implicit bias [1,31]. If experiencing burnout, women physicians experience emotional exhaustion first, which differs from male physicians [42]; women physicians are more frequently diagnosed with depression compared to male physicians [43], and they may have higher rates of suicide than male physicians when compared to their gender-matched general population [44].

### Study Aims

There is a growing need for best practices at individual, institutional, and systemic levels for overcoming physician gender inequity. We explore the experiences of women physicians who self-identify as being in the process of transitioning or who recently transitioned from early to mid-career to generate ideas that can inform detailed best practices for overcoming gender inequity during this time of career advancement. In this study, the aims are (1) to learn from the experiences and perspectives of women physicians as they transition from early to mid-career and (2) to develop best practices that can inform institutions and organizations in which women physicians work, to better support their advancement through an important transition from the early to mid-career phase.

## Methods

### Recruitment

We used qualitative methods using one-on-one semistructured interviews with women physicians. Participants will be identified through social media posts with invitations (eg, Facebook groups for Women Physician Entrepreneurs, Women Physician Leaders, etc) and through professional networks, listservs, or discussion forums to which team members belong. Eligibility criteria included self-identification as a woman who is in the process of transitioning or who has recently transitioned from early to mid-career. Purposeful sampling facilitated identification of information-rich cases, with attention to diversity in career pathway (eg, clinical, research, education, executive leadership, entrepreneurship, advocacy), specialty, and race and ethnicity [45]. To facilitate the recruitment process, women physicians could read a brief study description, including ethics review board approval, and voluntarily provide responses to a few initial screening questions (Textbox 1).

Snowball sampling was also intended to identify additional interview participants to enrich the sampled population if needed. Each participant will receive a US $100 Amazon gift card for their participation. We aimed to recruit approximately 20 women physicians for initial interviews. We anticipated the interviews to be approximately 1 hour in length.

**Initial screening questions and response options.**

1. In which US state or territory do you practice? (Response options were classified into 1 of 8 United States Bureau of Economic Analysis regions as part of the participant deidentification process)

2. What is your specialty? Check all that apply.

- Emergency Medicine
- Family Medicine
- General Surgery
- Internal Medicine
- Pediatrics
- Psychiatry
- Obstetrics/Gynecology
- Surgical subspecialties
- Other (please specify)

3. How do you describe your work environment(s)? Check all that apply.

- Academia
- Government or military
- Industry
- Private practice (eg, self-employed individually or in a group)

4. With which statement do you identify? Select one.

- I am in the process of transitioning from early to mid-career.
- I just completed my transition from early to mid-career.
- I’m not sure

### Study Procedures

An interview guide was developed by the investigators, who have diverse experiences in academic medicine (TIL, KHW, TLL, GTG, SSP, CYAC), health care administration (SSP), organized medicine (TIL, TLL, CYAC), private practice (TLL), and research (TIL, KHW, GTG, CYAC), including qualitative methods (KHW) (Multimedia Appendix 1). Qualitative interviews were performed via Skype or other acceptable web conferencing software by at least one investigator and recorded for transcription and analysis. Using an open coding approach, we analyzed transcripts using the constant comparative method, developed a code structure in stages in accordance with the grounded theory approach, and developed concepts through memoing [46-48]. Interviews continued until thematic saturation was reached. We plan to conduct participant verification with our original study participants.

### Ethics Approval

Study approval was received from the Ethics Review Committee of the Faculty of Health, Medicine, and Life Sciences (FHML-REC) at Maastricht University (FHML-REC/2019/056). Expedited review and approval was received from Stanford University (eProtocol #53654). The study was certified as exempt from review by the University of California, San Diego (Project #200463XX). Ethics approval was not sought from affiliate institutions for two authors (SSP, KHW) as neither author would interact with study participants and therefore would not interact with deidentified data at any time during the study. The study was randomly selected by the FHML-REC at Maastricht University for a research quality audit in October 2020, which was completed to the satisfaction of the Deputy Chair/Research Quality Officer of the FHML-REC.

Participants were informed about the study (the aim, method, data management, and the participants’ rights). Specifically, it was noted that some participants may experience psychological distress when remembering and recalling past experiences during one-on-one interviews, especially those that may involve particularly difficult or stressful emotions or memories. However, this was anticipated to be temporary and no harms are anticipated in association with participation in this study. Furthermore, participation was completely voluntary, and participants may withdraw from the study at any time. The researchers ensured that the consent was voluntary. Prior to interviews, the informed consent letter (Multimedia Appendix 2) and information sheet (Multimedia Appendix 3) were reviewed with participants.

### Data Management Plan

For participants who gave written informed consent prior to the interviews and therefore provided their name on the informed consent form, pseudonyms or pseudoinformation were used in the transcriptions to refer to the participants; it was not to be reported which names belonged to each transcript.

Only the primary research team members have access to the data. We established a monthly team meeting schedule to allow for discussion of concerns within the team. Data were anonymized by the primary investigator (TIL) throughout internal and external documents during analysis to remove identifiable personal and institutional information. For example, individual and institutional names, locations, and similar information were replaced with pseudoinformation (eg, an individual name would be substituted with XXX; the name of an academic institution would be replaced with “a large academic medical center”).

Data collected during the study are handled confidentially according to the European General Data Protection Regulation (GDPR). All the data are stored according to the rules of data management of Maastricht University (UM) [49]. We used the UM data archiving facilities and procedures to store our data. The data will be retained for 10 years, in accordance with the UM Data Management Code of Conduct. A data transfer and use agreement for anonymized data was established between Maastricht University and Stanford University to be in compliance with data management requirements of Stanford University as an affiliate institution of one of the authors (CYAC).

## Results

Twelve in-depth interviews of 50-100 minutes in duration per interview were completed and transcribed. Analysis of the data is expected to be completed by June 2022. Participant demographics of women physicians interviewed are supplied in Table 1.

In preliminary analyses, one key emerging theme is a tension resulting from finite time divided between demands from a physician career and demands from family needs. In turn, this results in constant boundary control between these life domains that are inextricable and seemingly competing against each other within a finite space; family needs impinge on planned career goals, if the boundary between them is not carefully managed. To remedy this, women sought resources to help them redistribute home responsibilities, freeing themselves to have more time, especially for children.

**Table 1:** Participant demographic characteristics.

Participant	With which statement do you identify?	How do you describe your work environment(s)?	Clinical specialty	US region^a^
1	I am in the process of transitioning from early to mid-career.	Academia	Anesthesiology	Far West^b^
2	I am in the process of transitioning from early to mid-career.	Academia; government/military	Internal Medicine	Far West^b^
3	I am in the process of transitioning from early to mid-career.	Government/military	Pediatrics	Far West^b^
4	I am in the process of transitioning from early to mid-career.	Private practice	Surgical subspecialty	Great Lakes^c^
5	I am in the process of transitioning from early to mid-career.	Private practice	Surgical subspecialty	Southwest^d^
6	I am in the process of transitioning from early to mid-career.	Academia	Emergency Medicine	Mideast^e^
7	I just completed my transition from early to mid-career.	Academia	General Surgery/Surgical Oncology	Plains^f^
8	I just completed my transition from early to mid-career.	Academia; government/military	Internal Medicine	Far West^b^
9	I just completed my transition from early to mid-career.	Private practice	Endocrinology	Southeast^g^
10	I’m not sure	Academia	Family Medicine	Far West^b^
11	I’m not sure	Academia	Internal Medicine	Far West^b^
12	I’m not sure	Private practice	Hospice and Palliative Care	Mideast^e^

^a^United States Bureau of Economic Analysis classifications of 8 US regions.

^b^Far West region includes Alaska, California, Hawaii, Nevada, Oregon, and Washington.

^c^Great Lakes region includes Illinois, Indiana, Ohio, Michigan, and Wisconsin.

^d^Southwest region includes Arizona, New Mexico, Oklahoma, and Texas.

^e^Mideast region includes Delaware, Maryland, Pennsylvania, New Jersey, and New York.

^f^Plains region includes Iowa, Kansas, Minnesota, Montana, Nebraska, North Dakota, and South Dakota.

^g^Southeast region includes Alabama, Arkansas, Florida, Kentucky, Louisiana, Georgia, Mississippi, North Carolina, South Carolina, Tennessee, Virginia, and West Virginia.

Women similarly sought resources to help with career advancement, although not with regard to time directly, but to first address foundational knowledge gaps about career milestones and how to achieve them. Such resources could be institutional professional development programs and/or relationship-based resources like having knowledgeable mentors. Effective mentorship was especially vital, mediating the translation of knowledge into prioritizing professional activities toward a previously unknown time horizon.

## Discussion

### Principal Results

Best practices for supporting the early to mid-career transition for women physicians are needed to overcome physician gender inequity. However, women physicians express uncertainty even in self-identifying as going through or completing this transition. The absence of clear signposts of career advancement, combined with variable levels of professional support, and tension generated at the boundary of professional and family life domains, leads to persistent challenges for and limited opportunities to support women physicians while they are navigating this career transition.

Upon completion of data analyses, we anticipate that the development of a set of best practices or at least guiding principles can be derived from the detailed, systematic collection of women physicians’ experiences. This may offer an impactful contribution to support women physicians as they advance through their career paths and address issues that arise in the leaky career pipeline. Due to the diverse and multifactorial contexts that women physicians may encounter both at work and at home, learning from the narratives of women physicians would be constructive toward the end goal of addressing such barriers for women.

A strength of this study is that it sought to include not only women physicians in academic medicine or in a single specialty but also to include women physicians in private practice in multiple US geographic locations and in different specialties. Despite this purposeful heterogeneity of the research participants, common themes appear to be emerging from the data, which will be analyzed and published in a future manuscript.

### Limitations

First, the recruitment for this study was limited due to difficulties that arose during the COVID-19 pandemic. Initial recruitment for interviews could only begin after multiple institutional review board approvals and data agreements could be obtained given the international collaboration of the coauthorship. By the time these approvals were obtained, the recruitment period coincided with the onset of the first global surges of the COVID-19 pandemic. The target population for recruitment included US women physicians, many who provided either frontline clinical care for patients during the first and subsequent surges of the COVID-19 pandemic or were otherwise engaged in pandemic responses.

Second, the parallel recruitment and interviews that occurred alongside pandemic responses in 2020 and 2021 may have affected the content and responses of participants recruited. Furthermore, this affected the content of the semistructured interviews, and may limit the generalizability of the results outside of the setting of the pandemic. Finally, recruitment was limited to US women physicians, which may limit the generalizability of results and best practice recommendations generated. Although the recruitment procedures were designed to permit inclusion of a diversity of women physicians by work environment, specialty, and geography, those women physicians who responded to the initial screening questions did not include any women physicians who work in industry or who are located in two US regions. The two regions not represented were New England region (Connecticut, Maine, New Hampshire, Rhode Island, and Vermont) and Rocky Mountain region (Colorado, Idaho, Montana, Utah, and Wyoming). Although the sample for this study is limited to the United States, women physicians globally experience gender inequity in career advancement and attrition from the practicing and academic physician workforce [4,5,50]. Future investigation could involve a broader diversity of women physician participants globally to examine additional influences on the early to mid-career transition, such as health care or academic systems and sociocultural considerations and the intersectional experiences of these physicians. Additionally, investigation of within-gender or between-gender differences, for example, by interviewing physicians who are men in the early to mid-career transition period, could offer additional insights about this career transition. There is also increasing acknowledgment that, upon transitioning into mid-career, systemic inequities lead to an “invisibility” of these women physicians as they seek continued career advancement [51]. Further work to mitigate these effects is also needed.

### Comparison With Prior Work

The preliminary results of this study are consistent with previous work in this area that examine issues of work-family conflict for women professionals. Work-family tension is encountered when demands from one domain are incompatible with the demands of another domain [52]. Although this study focused on women physicians only, the findings appear consistent with previous work that found that women professionals fit a Family profile compared to men who more commonly fit a Work profile [53]. A Family profile involves high importance given to family, including parental and spousal roles, whereas a Work profile involves high importance given to the work role. In another study, women and men who are junior faculty in an academic medical center were similar in their levels of career interest and career identification [54]. Although this study did not quantitatively assess women physicians’ career interest or work commitment, participants expressed an essential need to resolve work-family conflicts by finding personal and professional solutions to manage the boundary between each domain or set of roles.

Although this study was not originally designed to investigate the effects of the COVID-19 pandemic on women physicians and their career advancement, the effects of this major global event cannot be ignored. Several studies during the pandemic have sought to quantify the negative impacts of the pandemic on women physicians, especially those in academia including those in scientific research. For example, women as first authors on peer-reviewed publications decreased by 14% from 2019 (the year immediately prior to the onset of the pandemic) to 2020 (the first year of the pandemic) [55]. Additionally, concerns regarding women physicians taking on a disproportionately higher burden of family or caregiving responsibilities during the pandemic may influence their career transitions and advancement [56,57]. Preliminary findings of this study appear to be consistent with these observations, although this study may not have explicitly addressed these topics. Such topics also may have been less relevant for women physicians interviewed who were not in academia. Health care organizations, including academic medical centers, may be able to role model organizational professionalism and ethics, engage in benchmarking and reporting of gender equity data, and engage in meaningful interorganizational partnerships toward dismantling barriers to systematic gender inequities among physicians [58].

### Dissemination Plan

Findings will be summarized and described in detail in a manuscript and presented at national and international conferences as available. Findings will be submitted for publication to a peer-reviewed, open-access medical journal, due to the topic’s general relevance for women physicians, physician well-being, and health care organizations. Speaking engagements are expected, such as at an annual meeting for the American Medical Association’s Women Physicians Section (see Acknowledgments) and other professional societies or venues [59].

### Conclusions

Preliminary results provide initial insights about how women identified or activated a career shift and how they marshaled resources and support to navigate barriers they faced. However, further collaborative research and programs are needed to satisfactorily develop and implement best practices to support women physicians’ advancement through an important transition from their early to mid-career.

## Appendix

Multimedia Appendix 1 Interview guide.

Multimedia Appendix 2 Informed consent letter for research participants.

Multimedia Appendix 3 Information sheet for research participants.

## Abbreviations

FHML-REC: Ethics Review Committee of the Faculty of Health, Medicine, and Life Sciences
GDPR: General Data Protection Regulation
UM: Maastricht University (Universiteit Maastricht)
