# Supplementary material for: Women Physicians in Transition Learning to Navigate the Pipeline from Early to Mid-Career: Protocol for a Qualitative Study
Source: JMIR Res Protoc. 2022 Jun 2;11(6):e38126. doi: 10.2196/38126 (PMC9204597; doi:10.2196/38126)
Supplement: Multimedia Appendix 1 [file resprot_v11i6e38126_app1.docx]

*Thank you for participating in this study on women physicians in transition from early to mid-career. In participating in this study, you self-identified as being in early to mid-career transition.*

*I’d like to ask a few initial questions:*

- **How would you describe your workplace setting** (private practice/solo/small group; large group, outpatient/inpatient, academic, administration, research, etc.)**? What are your roles in your workplace, and how much time do you devote to each of your roles?**
- **What do you think are signs of the transition from early to mid-career?**
  - Probe:
    - How would you define early career? How would you define mid-career?
    - Were these definitions you derived or were they explained to you at some point in your career or training?
    - And where do you see yourself along this continuum from early to mid career?

*Please reflect on your experiences on the transition from early to mid-career.*

- **What decisions did you make (or are you making) that are key moments in your career?**
  - Probe:
    - What were pivotal moments in your career?
    - How would you describe the milestones for you from early to mid-career as you have made the transition? Were they positive or negative?
    - Can you tell me more about that?
    - Are there additional milestones you feel you need to reach before your transition is complete?
    - What are there events in your transition from early to mid-career that stand out in your mind?
- **How did *demands* on your time/energy (professionally and personally) change as you transitioned through your career?**
  - - Probe: Describe in what way they changed if they did.
  - **How did you prepare to meet those changes in demands?**
    - Probe: Did you feel prepared to meet those changes in time and energy demands?
  - **What *resources* do you find helpful to meet those demands?** In the workplace? At home? From somewhere else?
    - Probe: (As a resource) Did you have a role model, a mentor, or additional sources of support? If so, how did you find those sources of support?

*The next questions relate to work-life integration.*

- **How would you describe your non-work roles and how much time do you devote to those roles?**
- **Would you characterize your career this far as linear or non-linear?**
  - Probe: How would you describe the arc of your career pathway up to this point?
  - If non-linear, please describe any relevant details such as time off, unanticipated career shifts, re-locations. (Consider using as a lens for data analysis)
- **Do you feel your personal journey, your upbringing or the way you were raised influence how you have managed your professional life?**

*As we close the interview:*

- **How would you summarize what you have learned so far from this early to mid career transition process?**
  - Additional question: What is one piece of advice you would like to share with others about your journey?
- **(In closing) How has gender has influenced your career transition experiences?**

*This is the end of the interview. Thank you for your participation! I’d like to confirm your contact information for each of the following purposes:*

| YES/NO | $100 Amazon e-gift card. |
| --- | --- |
| YES/NO | Permission to follow-up for the “member check” step of the study? Results of our team’s analysis may be checked and further clarified with her to ensure alignment with her experience and our interview. |
| YES/NO | Interest in participating as an interviewee in a podcast series to share tips and stories about the experience of this transition (voluntary with no further compensation available). If so, then only her name and email will be shared with a producer from the podcast team (a person who is not involved with the podcast production and will have no knowledge of participation in the research study). |
| YES/NO | Interest in receiving follow-up email(s) about relevant publications or presentations based on the findings from the study. |
