# Supplementary material for: Women Physicians in Transition Learning to Navigate the Pipeline from Early to Mid-Career: Protocol for a Qualitative Study
Source: JMIR Res Protoc. 2022 Jun 2;11(6):e38126. doi: 10.2196/38126 (PMC9204597; doi:10.2196/38126)
Supplement: Multimedia Appendix 2 [file resprot_v11i6e38126_app2.docx]

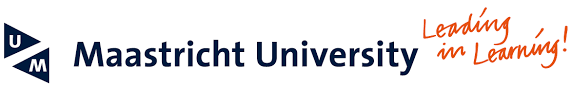
**Declaration of voluntary participation in research**

| **Project Title** | Women Physicians in Transition: Learning to Navigate the Pipeline from Early to Mid-Career | |
| --- | --- | --- |
| **Name and Address** | Maastricht University  Faculty of Health, Medicine & Life Sciences  Post address: Postbus 5800 \| 6202 AZ Maastricht  The Netherlands | |
| **Researchers** | Tiffany I. Leung, MD, MPH  Sima S. Pendharkar, MD, MPH  Chwen-Yuen Angie Chen, MD  Tammy L. Lin, MD, MPH  Geneen T. Gin, DO  Karen H. Wang, MD, MHS | |
| This research project has been reviewed and approved by the Ethical Review Board of the Faculty of Health, Medicine, and Life Sciences at Maastricht University. | | |
| The aims and methods of this research project, including information about data management and confidentiality, are described in the accompanying letter of informed consent. | | |
| **Declaration**   - I declare that I have been adequately informed about the research study and that I understand the information provided. - I give permission for my data to be stored for 10 years after the date of the last publication, in accordance with the Maastricht University Data Management Code of Conduct. - I am aware that research data collected is to be used for (inter)national scientific articles. - I am aware that I may terminate, for any reason and at any time, my participation in this research study. - I am prepared to participate in this research study. | | |
| **Name:** | | |
| **Signature (Participant):** | | **Date:** |
| By signing here, the researcher declares that the above-named volunteer is fully informed about the above-described research. | | |
| **Name:** | | |
| **Signature (Researcher):** | | **Date:** |
