# Supplementary material for: Women Physicians in Transition Learning to Navigate the Pipeline from Early to Mid-Career: Protocol for a Qualitative Study
Source: JMIR Res Protoc. 2022 Jun 2;11(6):e38126. doi: 10.2196/38126 (PMC9204597; doi:10.2196/38126)
Supplement: Multimedia Appendix 3 [file resprot_v11i6e38126_app3.docx]

**Project: Women Physicians in Transition: Learning to Navigate the Pipeline from Early to Mid-Career**

Dear Doctor,

Thank you for your interest in participating in our research project! In this letter is key information about the project, so please take a moment to review and ask any questions you may have before we proceed.

**What is the project about?**

This project consists of one-on-one interviews with women physicians who self-identify as transitioning from early to mid-career, or who identify as having recently completed this transition. You’re receiving this letter because you expressed that you may fit this category. We would like to hear about your experiences in this transition as the focus of this research. Each interview is expected to last about one hour.

**What happens with the information collected?**

The aim of this project is to collect information that will inform best practices for addressing challenges unique to this period of career transition. The [Maastricht University (UM) Data Management Code of Conduct](https://www.maastrichtuniversity.nl/research/integrity-ethics/management-research-data) (in English on pp. 4-5) will be strictly adhered to during the work. Thus, the findings of this study will be published in a scientific article, however, identifiable information will never be mentioned or shared.

**What are the conditions and other considerations for participation?**

- Participation is voluntary. If you decline to participate, or choose to terminate the interview prior to completion, then there are no repercussions.
- At the completion of an interview, participants will be offered a $100 Amazon gift card as compensation for their participants. Contact information collected to disburse the gift card will be used for *only* that purpose and then discarded.
- Interviews will be conducted by telephone, video conference, or in-person; 1-2 researchers from the investigator team will be present per interview.
- Interviews will be recorded to facilitate transcription by the primary investigator, in preparation for analysis.
- The primary investigator will anonymize the transcribed data prior to analysis.
- The investigator team will have access to data for analysis.
- All physical documentation, including signed informed consent, will be stored in a secure, locked cabinet, in an office only accessible by department-issued key held by the primary investigator.
- All digital documentation will be stored on a password-protected secure university server.
- Data collected will be stored securely for a 10-year period after the date of the last publication, in accordance with the UM Data Management Code of Conduct.

If you still have questions after reviewing this letter, then please contact Tiffany Leung via e-mail ([t.leung@maastrichtuniversity.nl](mailto:t.leung@maastrichtuniversity.nl)).
